# Azithromycin-Induced Bradycardia

**DOI:** 10.7759/cureus.16995

**Published:** 2021-08-08

**Authors:** Basel Abdelazeem, Rachel M Hollander, Sarah Ayad, Kirolos Gergis, Mohamed E Gismalla

**Affiliations:** 1 Internal Medicine, McLaren Health Care/Michigan State University (MSU), Flint, USA; 2 Internal Medicine, Michigan State University, College of Human Medicine, Flint, USA; 3 Internal Medicine, Rutgers-New Jersey Medical School/Trinitas Regional Medical Center, Elizabeth, USA; 4 Internal Medicine, McLaren Health Care, Flint, USA

**Keywords:** azithromycin, macrolide, bradycardia, bradyarrhythmia, case report

## Abstract

Azithromycin is a broad-spectrum antibiotic of the macrolide class and has multiple effects on the cardiovascular system, including prolonged corrected QT (QTc) interval. However, there is limited literature on the association between azithromycin and bradyarrhythmias. Monitoring the patient via telemetry can detect bradycardia. The diagnosis of azithromycin-induced bradycardia is usually made by the exclusion of other causes of bradycardia. We report a case of a 44-year-old female with past medical history of obstructive sleep apnea who presented to our hospital due to polysubstance drug overdose with possible aspiration pneumonia. The patient received azithromycin and subsequently developed symptomatic bradycardia two days post-onset of antibiotic treatment. This case raises awareness amongst physicians about the possibility of azithromycin-induced bradycardia and explains the different mechanisms that can cause it.

## Introduction

Macrolide antibiotics have multiple uses, including respiratory, urogenital, and other infectious etiologies [1,2]. It is considered a generally safe medication. However, few adverse reactions have been reported including gastrointestinal symptoms, headache, dizziness, and prolonged corrected QT (QTc) interval [3,4]. We report a case of a 45-year-old female who received azithromycin for aspiration pneumonia management and subsequently developed symptomatic bradycardia two days after the initial medication dose. This case highlights bradycardia as a potential side effect of azithromycin and the different mechanisms of azithromycin-induced bradycardia.

## Case presentation

**Figure 1:**
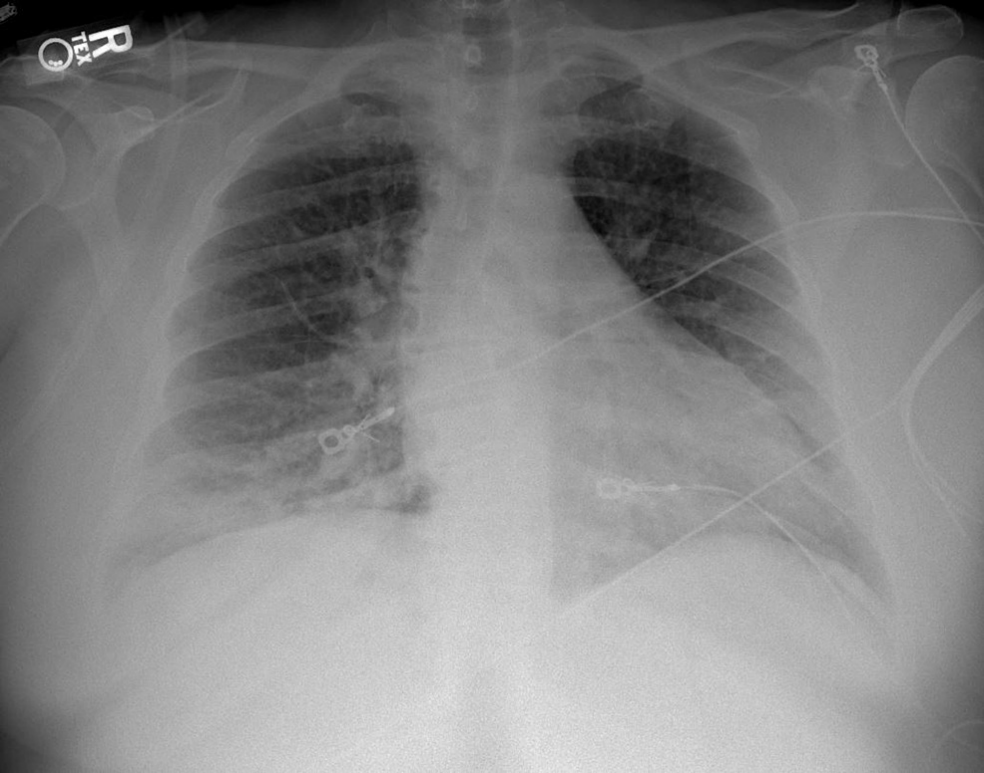
Chest X-ray. Chest X-ray revealed patchy airspace opacities mainly in the right middle and lower zones.

**Figure 2:**
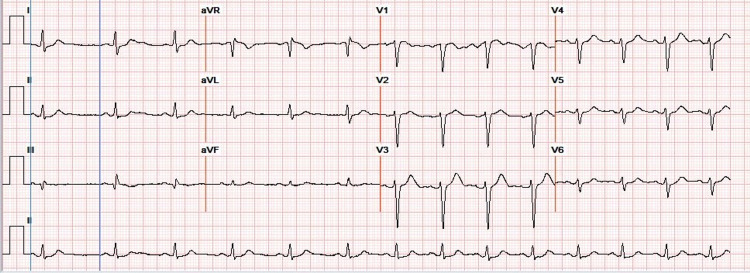
EKG on admission. EKG on admission revealed normal sinus rhythm with a heart rate of 81 and QTc interval of 410 msec.

A 44-year-old Caucasian female with a past medical history of obstructive sleep apnea (OSA), essential hypertension, type II diabetes mellitus, polysubstance abuse presented to the emergency department due to cocaine and heroin overdose. She was found unresponsive and required 8 mg of Narcan to regain alertness. The patient reported using continuous positive airway pressure (CPAP) at night, and she was compliant with it. In addition, the patient denied taking any beta-blocker or calcium channel blocker, and she also denied any family history of heart disease or arrhythmias. Initial vital signs in the emergency department include a blood pressure of 129/70 mmHg, heart rate of 98 beats per minute, oxygen saturation of 89% on room air, and respiratory rate of 22. On physical exam, the patient had regular S1 and S2, no murmurs, rubs, or gallops. Bilateral equal breathing sounds were heard without wheezing or crackles, and the patient’s BMI was 33.9. Laboratory workup revealed WBC 23.34 (4.50-11.00 X 10^3^/uL) and elevated lactic acid of 4.2 (0.5-2.0 mM/L). Chest X-ray revealed patchy airspace opacities, mainly in the right middle and lower zones (Figure 1). An electrocardiogram on admission showed sinus rhythm (Figure 2). She was started on 10 L oxygen via nasal cannula. The patient was started on azithromycin 500 mg once daily, flagyl 500 mg once daily, ceftriaxone 1g once daily for antibiotic coverage as treatment of aspiration pneumonia, and the patient was admitted to the medical floor.

On day 2 of hospitalization, the patient was bradycardic with a heart rate of 50 beats per minute with multiple episodes of bradycardia with a heart rate between 30 and 40 beats per minute on telemetry. The patient denied any symptoms associated with bradycardia, and she was transferred to the telemetry floor for closer monitoring. The patient denied any a history of bradycardia, and her previous echocardiogram two years prior demonstrated an ejection fraction of 55%-60% with no significant valvular deformities. The patient had was still on 500 mg IV of azithromycin till this point. TSH was 1.79 (0.350-5.500 uIU/mL). An echocardiogram completed after the onset of bradycardia showed an ejection fraction of 55-60% with mildly increased right atrial size (Video 1).

**Video 1:** Echocardiogram. Echocardiogram showing normal ejection fraction of 55%-60%.

**Figure 3:**
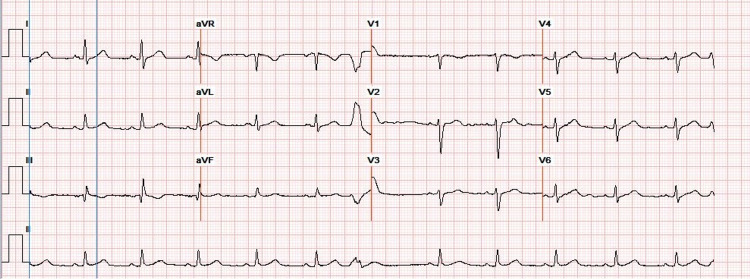
EKG after transferring the patient to CCU EKG after transferring the patient to CCU revealed premature ventricular contraction with a QTc interval of 427 msec.

Subsequently, on day three of admission, the patient developed symptoms of bradycardia, including diaphoresis, dizziness, lightheadedness, dyspnea, and chest pain. Telemetry revealed bradycardia with sinus pauses. The patient then received 1 mg of atropine, was initiated on a dopamine drip, and was transferred to the CCU. EKG revealed premature ventricular contraction with normal QTc interval (Figure 3). Her medication was carefully reviewed, and only azithromycin was discounted. The patient’s symptoms resolved 24 hours later, and we discounted the dopamine drip. However, the patient still had a few episodes of asymptomatic bradycardia ranging from 48-59 bpm. She had no current symptoms associated with bradycardia. The patient was discharged home with instructions to follow up with her cardiologist and a referral to nocturnal polysomnography to re-evaluate the OSA.

## Discussion

This case presents azithromycin-induced bradycardia in a patient with no previous history of episodes of bradycardia or other forms of arrhythmia. Bradycardia is a potentially life-threatening event. Multiple case reports reported azithromycin-associated QTc Interval prolongation in the literature, and only a few reported azithromycin-induced bradycardia with normal QTc interval [1,3].

The mechanism of azithromycin-induced bradycardia is still controversial. Macrolides are metabolized through the CYP3A4 isoenzyme and concomitant prescription of a macrolide with medications known to be inhibitors of the CYP450 system, increasing the likelihood of cardiac adverse effects with macrolide use, including prolonged QTc, bradycardia, and AV block [5,6]. Macrolide-induced bradycardia may also be dose-dependent, and the bradycardia is noted in higher doses mainly [7]. Another mechanism for macrolide-associated bradycardia is the potential unmasking of SA or AV nodal disease [3,8]. Furthermore, azithromycin can increase intracellular Na+ leading to calcium overload and polymorphic ventricular tachycardia [8].

OSA, especially if untreated, is a known cause of aberrant rhythms, including bradycardia. This pathophysiology is potentially related to OSA-induced hypoxia resulting in hypertension with reflexive bradycardia [9]. Our patient was provided with a CPAP machine at the hospital and had no previous history of bradycardia. The patient was not receiving any other medications that induce bradycardia and is not taking medications that are potent inhibitors of the CYP450 system. To the best of our knowledge, OSA played an exacerbatory role in azithromycin-associated bradycardia in our patient, and no known reports have previously demonstrated an association with OSA, azithromycin use, and bradycardia with normal QTc interval.

We recommend that physicians should be cautious when prescribing azithromycin. It is essential to understand a patient’s risk of bradycardia before starting azithromycin and considering starting azithromycin while the patient is on telemetry for closer monitoring, especially in patients with a family history of cardiac arrhythmias like long QT syndrome [4], electrolyte abnormalities like hypokalemia [2], patient on antiarrhythmic drugs [2] or concurrent usage of azithromycin and other medication that inhibit CYP450 like nifedipine should be avoided [5].

## Conclusions

Azithromycin is associated with cardiac side effects. QTc interval prolongation is a common side effect, but symptomatic bradyarrhythmia is a rare side effect. Before starting the patient on azithromycin, the physician should check if the patient is taking other medication that can inhibit CYP450 in the liver as concurrent usage can induce bradyarrhythmia. In addition, patients with underlying cardiovascular risk factors should be monitored while starting azithromycin. We present a case of a 44-year-old Caucasian female who had aspiration pneumonia and was started on azithromycin. The patient developed symptomatic bradycardia that was relieved after discontinuation of the azithromycin. This case highlights bradyarrhythmia as a side effect of azithromycin and the different mechanisms that can cause it.
